# First Results of a Detection Sensor for the Monitoring of Laying Hens Reared in a Commercial Organic Egg Production Farm Based on the Use of Infrared Technology

**DOI:** 10.3390/s16101757

**Published:** 2016-10-21

**Authors:** Mauro Zaninelli, Veronica Redaelli, Erica Tirloni, Cristian Bernardi, Vittorio Dell’Orto, Giovanni Savoini

**Affiliations:** 1 Università Telematica San Raffaele Roma, Via di Val Cannuta 247, Rome 00166, Italy; 2 Freelance Certified Infrared Thermal Technician, Oggiono (LC) 23848, Italy; vereda@tin.it; 3 Department of Health, Animal Science and Food Safety (VESPA), Università degli Studi di Milano, Via Celoria 10, Milan 20133, Italy; erica.tirloni@unimi.it (E.T.); cristian.bernardi@unimi.it (C.B.); vittorio.dellorto@unimi.it (V.D.); giovanni.savoini@unimi.it (G.S.)

**Keywords:** infrared sensors, laying hens, organic egg production systems, ozone

## Abstract

The development of a monitoring system to identify the presence of laying hens, in a closed room of a free-range commercial organic egg production farm, was the aim of this study. This monitoring system was based on the infrared (IR) technology and had, as final target, a possible reduction of atmospheric ammonia levels and bacterial load. Tests were carried out for three weeks and involved 7 ISA (Institut de Sélection Animale) brown laying hens. The first 5 days was used to set up the detection sensor, while the other 15 days were used to evaluate the accuracy of the resulting monitoring system, in terms of sensitivity and specificity. The setup procedure included the evaluation of different color background (CB) thresholds, used to discriminate the information contents of the thermographic images. At the end of this procedure, a CB threshold equal to an increase of 3 °C from the floor temperature was chosen, and a cutoff level of 196 colored pixels was identified as the threshold to use to classify a positive case. The results of field tests showed that the developed monitoring system reached a fine detection accuracy (sensitivity = 97.9% and specificity = 94.9%) and the IR technology proved to be a possible solution for the development of a detection sensor necessary to reach the scope of this study.

## 1. Introduction

### 1.1. Laying Hens Farming

Laying hens are an important sector within the intensive animal productions. Laying hens can be reared in enriched cages or in no-cage alternative housing systems. In these systems, animal welfare is improved because hens are able to move freely [1]. Furthermore, they often provide an outdoor area that reduces the density of hens in the house during the daytime, increases access to resources, and gives hens the opportunity to perform spatial behaviors [2].

Within those no-cage alternative housing systems, a specific case are systems used in commercial organic egg production farms. Their design depends on regulations that these farms generally share. These regulations typically require: (1) the adoption of an outdoor area, covered with grasses or plants, with a bigger size per hen; and (2) the rearing of flocks of no more than 500 hens. As a result, many hens are encouraged to use the outdoor area. Even though its use depends on many factors—such as temperature, wind, precipitation, season, and age—it has been observed that in these systems, under some specific circumstances, the mean number of hens that use the outdoor area can be up to 35%–40% of the whole flock [2]. Furthermore, adopting some specific management practices (such as a limitation of feed before the opening of the outdoor area), using a covered outdoor area with feeders and specific devices, such as pop-holes that can be set up as a “one-way passage”, the quota of hens that use the outdoor area could be incremented and may enforce the inclusion of the whole flock for a limited time of the day.

### 1.2. The Management of Housing Systems for Laying Hens

The management of housing systems is crucial to guarantee hens’ welfare and farm productivity. Many parameters have to be controlled and maintained in specific ranges. Temperature should move in a range from 16 to 24 °C [3]; relative humidity should be kept between 50% to 70% [3]; the level of atmospheric ammonia, mainly due by the microbial population that is present in the poultry litter [4], should not exceed 20–25 ppm for a period of time of no more than 8 h [5]; the level of carbon dioxide, mainly due by the presence of heating systems and of animals [6], should not exceed 3000 ppm for no more than 8 h [7]; the level of dust concentration, that can vary in a range from 0.02 to circa 80 mg/m^3^ for inhalable dust and from 0.01 to 6.5 mg/m^3^ for respirable dust [8], on the basis of the type of housing system considered, should be reduced as much as possible. A way to control the above-cited parameters is to use a correct cycle, and intensity, of ventilation. This generally allows control of the temperature, reduction of the relative humidity—and consequently the levels of atmospheric ammonia and bacterial load—and improvement of the air quality [9]. Nevertheless, excessive ventilation can cause other kinds of problems. A much too low relative humidity can increase dust and produce high heating costs during cold periods [3]. Therefore, for a better management of housing systems for laying hens, other strategies, that could be added to those already used, could be useful.

Ozonization may be an interesting procedure to take into consideration. It is a sanification treatment that could improve air quality and reduce odors, levels of atmospheric ammonia, and bacterial load. Its use has been evaluated in many applications of the poultry industry [10,11,12] and also in intensive animal production units [13]. Its full effectiveness is not proved yet, and some possible issues regarding production and health—of both animals and human operators—are under evaluation. In detail, it is well known that for a bactericidal purpose, a level of ozone (in the range of 1 ppm and above) is required [14], but on the other hand, the toxicity of ozone is dose-responsive and depends on the level and time of exposition, for both animals and humans [15,16].

In commercial organic egg production farms, the use of ozone could be facilitated. Since hens could be in the outdoor area, higher concentrations could be used in order to achieve a better efficacy. At the same time, its toxicity could be avoided because the housing system could be opened to hens only when ozone concentrations would be not dangerous. However, this process should be controlled; a monitoring system and a detection sensor, able to identify the presence of animals in the housing system before the start of ozone treatments, would be necessary.

### 1.3. Monitoring Systems for Laying Hens

From a technical point of view, different technologies have been already evaluated in order to automatically detect the presence, and the movements, of laying hens inside a housing system. The most used, in behavioral studies of laying hens, is the radio frequency identification (RFID) [1,17,18,19,20]. This technology is based on the use of passive transponders (also called tag) that can be fastened to legs or wings of hens. These tags transmit a unique identification number when activated by an external electromagnetic field created by an antenna. The antenna is connected to a dedicated reader that uses 134.2 kHz or 125 kHz as operating frequencies in its communication protocols. The physical position of the antenna, inside the housing system, allows detection (and recording of) the presence of one hen in a specific place. If many antennas are used, more places can be controlled by the resulting monitoring system. These monitoring systems have been mainly used to carry out research studies on behavior of laying hens reared in alternative housing systems. Their use in production systems could be inappropriate for many reasons. For example: (1) all hens should have a tag for being identified and with a large flock, this requirement could be expensive and time consuming; (2) many antennas (and readers) should be used to monitor all the space inside a housing system and in the case of an inner space of only few square meters, this requirement could considerably increase costs and complexity of the structures; (3) during normal operational activities, tags could be lost by hens or stop of working, in these cases, to identify the hens without an operative tag and to manage the necessary substitution could be a difficult task.

Another technology that has been tested to automatically detect laying hens is based on sensors that allow the building of a geographic information system (GIS) [21,22,23]. These sensors have to be worn by hens. They are radio mobile sensors that allow the building of a wireless network. When coupled with stationary nodes, placed inside the housing system, they act as beacons for the proximity detection of hens and allow the collection of data about the presence and the movements of the animals. When compared with RFID technology, this solution seems to offer some advantages. For example, it is able to control a bigger area with a fewer hardware components. Nevertheless, also this technology appears to not be ideal for the detection of laying hens in a productive scenario. Since the sensors worn by hens have to perform active tasks, they need to use a power supply. This could be a limit for the development of monitoring systems that have to work for a long time. Furthermore, even though the number of hardware components are fewer than the RFID solution, their use on a large scale can always bring the development of expensive systems.

A further technology that has been considered for the automatic monitoring of laying hens is imaging analysis. In scientific literature, some examples can be found [3,24,25]. However, the aim of this kind of studies is generally to acquire the behavior of a group of animals. The resulting monitoring systems are not set up to detect a single hen and, consequently, a good accuracy cannot be guaranteed. The only example that can be taken into consideration, on the basis of our knowledge, is a monitoring system developed by Zaninelli et al. [26], which was designed to automatically detect a possible double occupation of hens in a nest of an alternative housing system. This system uses a commercial webcam that acquires images from the nest interior in the visible spectrum. Images are processed by a dedicated software application and an alarm is recorded in case a double occupation is detected during the period of time in which the nest is available for hens. The results obtained during the experiments showed that imaging analysis itself could be a useful technology for the automatic detection of one or more hens in a well-defined space. Nevertheless, some limits can be highlighted. For example, in order to achieve good accuracy, it was necessary to have the nest interior colored black in order to increase the contrast between the hen and the background. Furthermore, the sensor showed sensitivity to low light conditions and shadows that could affect the general accuracy of the resulting monitoring system.

A possible way to overcome the last cited issues could be the use of sensors that acquire digital images in the infrared spectrum (IR). This technology produces digital images where each spot represents, through its color, the temperature of the surface under evaluation [3]. Infrared technology has been used extensively for industrial, medicinal, and military applications because it allows acquisition of images in broad daylight or during the night, in all weather conditions, and is not affected by reflections of the light and/or shadows. It is a noninvasive technique and it has a high precision, especially on animal coats with low heat capacities [27]. In poultry research, IR technology has been investigated for the evaluation of the welfare of animals [24,28,29]. However, scientific papers on the use of this technology are few and many authors agree that further research would be useful in order to provide adequate information and to produce enhanced monitoring systems within poultry houses [3,28,29].

The development of a monitoring system that uses a detection sensor based on IR technology in order to identify the presence of laying hens in a closed room was the aim of this preliminary investigation. This monitoring system, in future, could allow application of ozone treatments—in organic eggs production farms—in a safe mode, and consequently achieve a possible reduction of levels of atmospheric ammonia and bacterial load.

## 2. Materials and Methods

### 2.1. Detection Sensor Layout

The detection sensor was mainly composed by a commercial thermographic camera (Thermo GEAR G120, AVIO, Nippon Avionics Co., Ltd., Tokyo, Japan) with a size of 21.2 cm (H) × 7.5 cm (W) × 13.8 cm (D), an uncooled focal plane array (microbolometer) detector with a resolution of 320 × 240 pixels, a sensitivity of 0.04 °C (at 30 °C), and an accuracy of ±2 °C or 2% of the operating temperature: 0 to 40 °C. The thermographic camera was connected through its analog video output to an analog/digital video acquisition board (Roxio, Santa Clara, CA, USA) that provided images acquired from the thermographic camera, in a digital format, to a specific software subroutine. For the developing of this software subroutine, NI Vision Development Module 2009 (National Instruments, Austin, TX, USA), NI Vision Acquisition Software 2009 and NI LabVIEW 8.5 were used.

During the experimental phases, the sensor software subroutine performed some specific elaborations on the images acquired by the thermographic camera. In details, a 2-dimensional array of colored pixels was built from each thermographic image and each colored pixel of the array was compared to a “color background” (CB). The total number of “colored pixels” (CP) was calculated by the comparisons performed and the resulting value was compared with a threshold called “hens detected” (HD). The HD threshold was chosen considering the most restrictive (i.e., the visible surface of one hen). Therefore, with this setup, the sensor was able to also detect cases in which more hens were together in the closed room. When the value of CP overcame the HD threshold, a monitoring system received a signal from the sensor software subroutine.

### 2.2. Experimental Monitoring System

**Figure 1:**
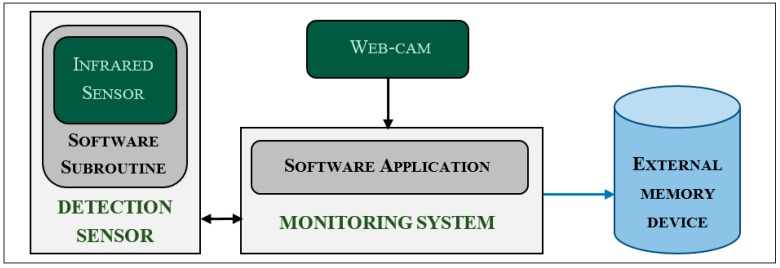
Block schema of the experimental monitoring system.

**Figure 2:**
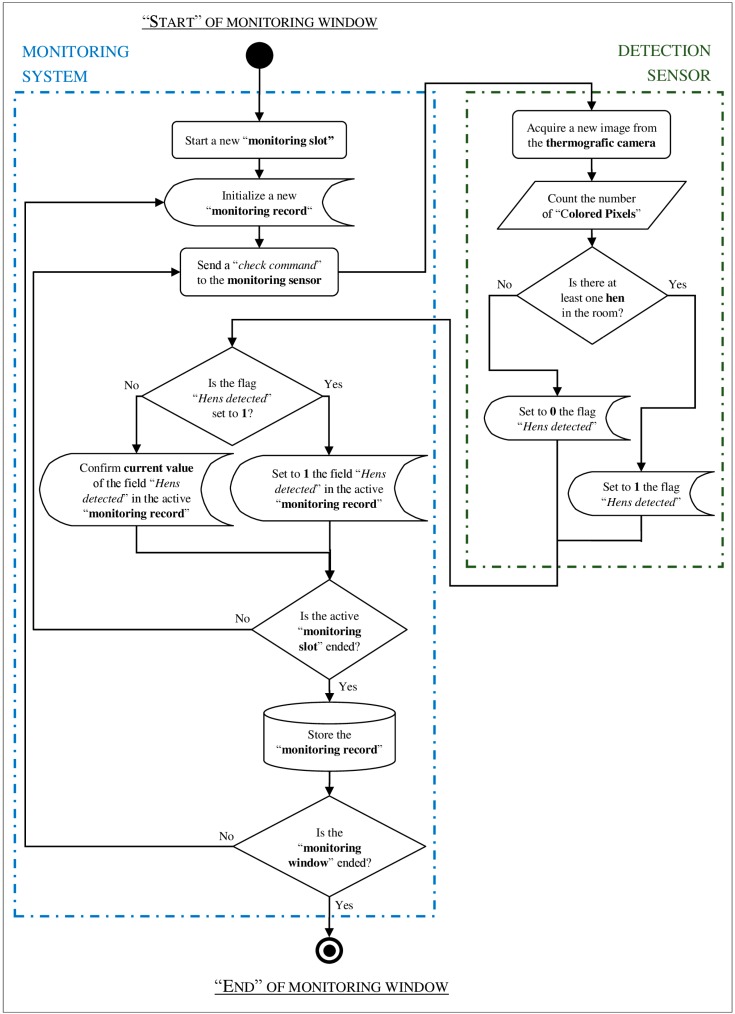
Flow diagram of the software applications of the monitoring system and detection sensor.

An experimental monitoring system, to which the detection sensor was connected, was used to set up the sensor and, in following steps of the experiment, to evaluate its accuracy. The system consisted of a dedicated software application (developed with NI LabVIEW 8.5—Figure 1) the algorithm of which is described in the flow diagram of Figure 2.

According to the diagram, when a “monitoring window” starts [30], a new “monitoring slot” is activated and a new “monitoring record” is initialized [31]. In a following step, the software application checks if at least one hen is detected, through the detection sensor, and in case of a positive result it updates the monitoring record. These checks continue, at a rate of one cycle every 2 s, until the “monitoring slot” is ended (i.e., for 20 s in order to allow the evaluation of 10 images for each single slot). At the end of the “monitoring slot” the current monitoring record is stored in an external memory device, through a text log-file, with the time stamps of the start and end on the slot, also including other data of the detection sensor setup such as the thresholds CB and HD. After this step, a new “monitoring slot” starts and a new monitoring record is initialized. This sequence of activities continues until a monitoring window is closed (i.e., the monitoring system is switched off).

The experimental monitoring system also stored images acquired by a commercial webcam (AC 150, HAMA, Hama Gmbh & Co., Monheim, Germany) installed close to the sensor developed (Figure 1). For each check of the monitoring sensor, one image was recorded (i.e., with a sampling interval of 2 s). All collected images were converted into single snapshots with a “.bmp” extension and a resolution of 320 × 240 pixels and used to build video recordings with an “.avi” extension, highly compressed by the filter “Microsoft Video 1”, and a picture sampling rate of 1 frame per second. Snapshots and video recordings were acquired every day from the start to the end of a monitoring window. Dates and time stamps were added to each image and used to name each file [32]. Images and video recordings were used in a preliminary phase of the experiment to set up the detection sensor and analyzed in the following phases to evaluate the accuracy of the monitoring system.

Although the thermographic camera used for the experiment was able to acquire snapshots in the visible spectrum, a commercial webcam was connected to the monitoring system. This technical solution was adopted because it was an easy way to collect images from the nest interior. Furthermore, not all commercial thermographic cameras provide the feature to collect images in the visible spectrum. This is the case of a “family” of commercial thermographic cameras specifically developed for industrial applications. They are generally compact, robust, less expensive, and allow transfer of images through a standard network and interface (GigE Vision), even over long distances, using low-cost standard cables. These cameras are ideal for monitoring applications. Nevertheless, they generally do not allow acquisition of images in the visible spectrum. The technical solution adopted in this study was chosen also for this reason. It could allow the use of this kind of thermographic cameras in possible future developments of the monitoring system under study.

### 2.3. Experimental Housing System

**Figure 3:**
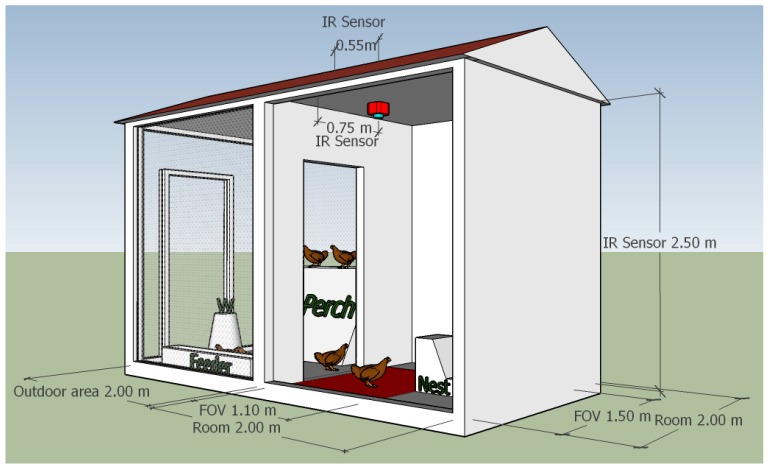
The picture reports the setup of the housing system. The water dispenser, that is represented with the symbol “W”, and the other main components of the system are reported. In the closed room, drawn in red, are shown: the infrared (IR) sensor (i.e., the thermographic camera) mounted on the ceiling of the room and the field of view (FOV) of the sensor highlighted on the floor of the room. Finally, dimensions of the housing system, of the IR sensor position, and of the FOV, are also reported.

In order to evaluate and set up the sensor developed, an experimental no-cage housing system was selected and used. It was a separate housing system of an organic eggs production farm, located in a region of Northern Italy (Lombardy), in which the laying hens reared were ISA brown (Institut de Sélection Animale). The housing system included a closed room of 2 m (H) × 2 m (W), a covered outdoor area of the same size, one nest (of 30 cm (W) × 45 cm (D) × 45 cm (H)), a feeder, a perch, and an appropriate water dispenser (Figure 3). The housing system provided 8 m^2^ of floor space while the outdoor area provided 0.57 m^2^ of floor space per hen since the flock used for the experimental phase involved a total number of 7 hens. This value was chosen after having considered articles 4.1.e, 4.1.c, and 4.4 of the EU Council Directive 1999/74/EC that defines the minimum standards for the protection of laying hens. The hens of the experimental group were randomly selected, and among the laying hens that were reared in the farm (already in a group), approximately at the age of 18 weeks, and housed in the experimental housing system a month before the start of the tests.

In the closed room, the thermographic camera was installed. It was positioned 2.5 m above the floor with its lens pointing directly downwards and immediately after the room entrance. Its field of view (FOV) was of circa 110 cm (H) × 150 cm (W). Therefore, during the image acquisitions performed, the floor space inside the closed room (of 2 m (H) × 2 m (W)) was partially limited to the hens of the experimental group in order to monitor all of the space available for the animals using the sensor under study. A webcam, connected to the monitoring system, was also installed in the room. It was positioned close to the thermographic camera and with the same setup in order to acquire images similar to those evaluated by the sensor during its monitoring activity.

### 2.4. Procedures for Data Collection and Processing

The tests were conducted for three weeks (from 16 July to 5 August). During the first 5 days, data for the setup of the developed sensor were collected while in the other 15 days, data for the evaluation of its accuracy were acquired.

In the days of sensor setup, a specific procedure was performed by a researcher. It required the execution of the following steps: (1) possible hens that were in the room were forced to get out; (2) one hen from the flock was randomly selected and moved inside the room; (3) the room was closed; and (4) the monitoring window was started (at 11.00 a.m.) in order to allow the monitoring system to acquire experimental data for 30 min. After that interval of time, the monitoring system was stopped and each limitation was removed. This procedure was performed in order to acquire enough data, for the sensor setup, in a well-controlled scenario (i.e., one hen in the room). On the other days of experiment, this procedure was not performed. Therefore, a researcher only switched on the monitoring system between 11.00 a.m. and 11.30 a.m., and he/she did not carry out any action on animals eventually inside the room.

During the days of sensor setup, the sensor software subroutine did not make any evaluation on images acquired. Furthermore, a dedicated version of the monitoring software application was used. Through this version, thermographic images were collected by the monitoring system and stored in an external memory device (with a “bpm” format), in addition to those already acquired from the webcam installed in the closed room. Finally, no monitoring records and/or text log-files were collected.

After the first days of data collection, some steps were performed to complete the setup procedure. In detail, a subset of thermographic images (n. 1500), within those collected (n. 4389), were evaluated through a version of the sensor software subroutine that was able to work off-line on “bmp” files stored in a folder. With these images, three thresholds of CB were investigated. They corresponded to an increment of 1, 3, and 5 °C from the floor temperature. These temperature shifts were chosen considering what scientific papers suggest [28,29,33,34] as possible ranges of body temperatures that should result from a thermographic image of a hen.

For each threshold of CB investigated, and thermographic image selected, the number of CP was calculated. As a result, three sets of data were obtained, and for each of them, the following analyses were performed:

- a Student’s *t*-Test (*t-*test procedure, of the package *stats* [35], of the statistical tool R [36]—version 3.3.0, 2016) in order to check the significance of the mean value and to identify a confidence interval at 95%.
- A receiver operating characteristic analysis (ROC) to evaluate, at different possible cutoff levels (i.e., the possible HD thresholds), the sensitivity, and specificity showed by the sensor developed.

In a further step of the procedure of sensor setup, a specific value of sensitivity was chosen (i.e., 80% because it was considered as a reasonable value of the human ability to decode a thermographic image), and all the corresponding specificities for the three thresholds of CB studied were found using the ROC curves built. In the experimental steps that are followed, the threshold of CB that was selected and used was the one that showed to reach the best result in terms of specificity. Furthermore, the corresponding cutoff level was found and set up as HD threshold.

In the following days, field tests were carried out. At the end of each “monitoring widow”, a researcher switched off the monitoring system and evaluated data acquired, comparing the log-file provided by the monitoring system and the images (and/or video recording) from the room interior. From these comparisons, he/she classified the results obtained by the monitoring system in terms of: true positive (TP), in the case of correct detection of one or more hens that were in the room; false positive (FP), in the case of wrong detection because no hens were in the room; true negative (TN), in the case of correct detection that no hens were in the room; and false negative (FN), when one or more hens were in the room and they were not detected by the sensor. At the end of the experiment, all these data were evaluated, and sensitivity and specificity calculated were considered as the detection accuracy achieved by the monitoring system under study.

During all these testing phases, in order to ensure that high quality images were been acquired by the thermographic camera and by the webcam, their lenses were checked daily and cleaned by a researcher.

## 3. Results

Different thresholds of CB were evaluated during the setup procedure of the detection sensor. The results that were obtained are shown in Table 1.

As reported in the table, and for each threshold of CB studied, a significant mean value was always found.

In a following step of the setup procedure, the sensitivity of the sensor was set to be at least 80% and for each threshold of CB investigated, the corresponding specificity was calculated. In Table 2, these specificities are shown.

As reported in the table, the threshold of CB that corresponded to an increment of 3 °C from the floor temperature, achieved the highest specificity. Starting from this result, and in the following days of the experiment, for the setup of the sensor subroutine the following parameters were used: a threshold of CB equal to an increase of three degrees from the floor temperature and a threshold of HD equal to 196 colored pixels.

As a final experimental step, the accuracy of the monitoring system was evaluated. Log-files provided by the monitoring system, and images (and/or video recordings) acquired from the room interior, were compared. Results that were obtained are shown in Table 3.

**Figure 4:**
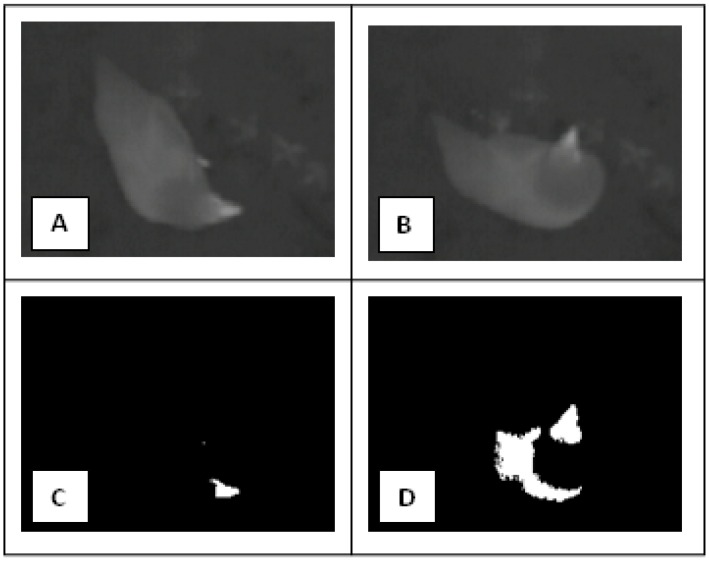
Pictures (**A**,**B**) are examples of the thermographic images collected during the experiment, while pictures (**C**,**D**) are examples of the elaborations performed by the monitoring system under study. In details, picture (**C**) is the result of the elaboration performed having as input the picture (**A**) while picture (**D**) is the result of the elaboration performed having as input the picture (**B**). In picture (**C**), the number of colored pixels (CP) is 113 while in picture (**D**), the number of CP is 1832. Therefore, considering a cutoff threshold of 196 pixels (“hens detected” threshold), picture (**C**) is an example of a false negative case while picture (**D**) is an example of a true positive case.

As reported in the table, the monitoring system achieved a sensitivity and specificity of: 97.9% and 94.9%. Furthermore, only in 18 cases was a hen in the room but was not detected by the system during a “monitoring slot” (Figure 4).

## 4. Discussion

This study was carried out in order to develop a system for the monitoring of laying hens in commercial organic egg production farms. Its specific aim was the development and testing of a sensor, based on infrared technology, able to detect the presence of hens in a closed room in order to start possible disinfectant treatments without affecting animals’ health. Obtained results have shown that the monitoring system developed could reach this goal since the achieved accuracy was a sensitivity of 97.9% and a specificity of 94.9%. These results were partially expected. They were mainly due by the adoption of a “monitoring slot” as a setup of the monitoring system tested. This setup allows evaluation of more than one image, through the detection sensor, before classifying a case. As a consequence, the accuracy of a detection sensor is generally improved and it can obtain useful results [26].

Nevertheless, some cases classified as false negatives occurred during the experiments. These cases were mainly due by three unfavorable events, which occurred in one or more “monitoring slots”, that were: (1) some hens were in the nest and therefore not detectable by the sensor; (2) some hens were in an area of the room not well managed by the sensor; and (3) the CP threshold was not adequate for the detection of a hen. However, some improvements could be done to the monitoring system tested in order to overcome these unfavorable events. For example, simple photocells could be installed in the nest of the housing system in order to detect the possible presence of a hen. These photocells could be connected to the monitoring system, overcoming these false negative cases. Furthermore, the FOV of the detection sensor was mainly limited by the sizes of the closed room that was not under our control. Nevertheless, we think, on the basis of our experience, that the thermographic camera could be mounted up to 3–5 m from the floor without losing the accuracy showed in this preliminary evaluation. Using a commercial wide-angle lens, with a focal length of 7.5 mm or 4 mm, the thermographic camera could show a bigger FOV (up to 90° (H) × 70° (V)) and with a detector of 640 × 480 pixels, the distance from the floor could be increased, allowing an almost doubling FOV. With this technical layout, the possible false negative cases due to an area not well managed by the sensor should be reduced. As an alternative, the detection sensor could be moved in the room. Rotating it around one axis or moving it in parallel, the sensor could make a scan of the floor. This solution could allow detection of a hen also in a bigger room than the one used in this experiment, and it shouldn’t affect the global efficiency of the monitoring system since, as suggested by our results, a “monitoring slot” of only 20 s could be enough to achieve a fine detection accuracy for each single area investigated. Results obtained also proved that the CP threshold, selected in the setup procedure, was correct in the majority of the cases evaluated by the detection sensor. Nevertheless, for a few number of “monitoring slots”, this threshold did not permit detection of a hen that was in the room. Nevertheless, all the false negative cases related to this unfavorable event principally occurred on one specific day, which was the hottest one of the weeks involved in the experiment (28 °C at 11.00 a.m., unlike in the setup days where temperatures ranged from 23–27 °C). This result seems to suggest that a CB threshold should not be selected as a constant increment from the current floor temperature but as an adaptive value on the basis of the absolute atmospheric temperature [36,37], at least when this temperature is outside from a common operational range of temperatures that could be defined for the monitoring system. Further investigations in other months of the year, and atmospheric temperatures, will be useful to deeper understand this fact and in case of the need, to develop an adaptive CB threshold. We think that this improvement could limit the false negative cases observed in this preliminary tests, and related to this phenomenon, allowing to achieve a better sensor detection accuracy. Finally, as a last possible improvement, we report that the “monitoring slot” could be always extended, or as alternative, the monitoring system could be set-up in order to evaluate more than one “monitoring slot” before classify a case. In our tests, all the hens that were not identified in a “monitoring slot” were, most of the times, correctly detected in the following “monitoring slot”. Therefore, a monitoring system set-up that evaluate at least two “monitoring slots” before classify a case should allow to overcome most of the false negative cases that were observed in this preliminary study.

During the testing phases, the lens of the thermographic camera was checked daily and cleaned by a researcher to ensure that high quality images were acquired. This could be considered as a limit of the monitoring system developed, because investigations in a time period longer than one day could be difficult. However, it is important to note that this system was mainly developed for commercial organic egg production farms where the sizes of the flocks are, in general, no more than 500 hens. Therefore, in these farms, the quality of the air inside the housing system is generally better than in the conventional farms. Nevertheless, some technical solutions to reduce this possible limitation could be identified. The level of dust, inside a housing system, is inversely related to the level of relative humidity. This parameter can be controlled by the cycles and intensity of ventilation. Therefore, a specific procedure, or specific corrections to the procedures already used, could be developed in order to limit the level of dust, also in cases of housing systems that use only a natural ventilation. Furthermore, a dedicated device that automatically clean lenses of the thermographic camera could be adopted. For example, a simple nozzle to spray compressed air could be mounted close to the thermographic camera in order to clean lenses before the start of the monitoring window, or in repeated intervals of time to prevent a deposition of dust. Finally, both these strategies could be combined and controlled by a dedicated air quality sensor in case a more articulate procedure would be necessary. Otherwise, a commercial pneumatic system for shielding thermographic cameras, able to open a small door only when the camera has to acquire images, could be always adopted. Further investigations will be useful to analyze this possible problem and to identify the right procedure and/or technical solution.

In general, obtained results proved that imaging analysis was an interesting approach to the aim of the present study, and infrared technology showed to be a valid means to detect the hens that were in the closed room. In fact, different light conditions or shadows did not affect the quality of acquired images. Furthermore, the availability of an IR camera in a housing system could be useful also for other purposes. Up to day, thermographic image analysis of poultry has proven to improve the welfare of these animals [19,20,22]. For example, Yahav et al. [33] evaluated data from an IR camera in order to measure the body surface temperature of chickens as a basis for calculating heat loss by radiation and convection. The authors found that IR technology allowed evaluation of the physiological status of the chicken under varied environmental conditions, permitting to improve climate-control systems, welfare, and performance of the flocks. Ferreiera et al. [28] investigated the effectiveness of IR thermography evaluating the loss of sensible heat in young broilers fed with different dietary energy levels. They found that an IR camera was able to record young broilers’ surface temperature variation, when birds were fed with different diets, allowing to increase productivity and animal welfare. Baracho et al. [29] used the IR spectrum in order to measure the surface temperature of broilers as a function of heat distribution inside broilers houses. They found important variations of skin-surface temperature due to week of grow-out and sector of the broilers house, demonstrating that the broilers were suffered a thermal distress. However, scientific papers that explored IR technology applied to poultry industry are still few, probably for the high initial costs of the necessary devices [3]. Therefore, the availability of a monitoring system already equipped with one or more IR cameras could be a stimulus for future researches. Furthermore, it could be a chance to scale up, at farm level, the available knowledge on this technology in order to realize an enhanced monitoring system, within poultry houses, that could better control, and so improve, the welfare of hens. This could be useful also for farmers since productivity should improve.

The future phases of this project will regard extensive tests of the monitoring system developed. Furthermore, the system will be completed with an ozone machine in order to also test the effectiveness of ozone treatments, having as final target of reaching a possible reduction of atmospheric ammonia levels and bacterial load in commercial organic egg production farms.

## 5. Conclusions

Obtained results suggest that the monitoring system developed could be able to identify the presence of laying hens in a closed room, using a detection sensor based on the IR technology. As a consequence, they prove that IR technology was a valid technical solution for the aim of this study. Further researches will be carried out in order to confirm these results, to complete this monitoring system and to evaluate the effectiveness of ozone treatments to reduce atmospheric ammonia levels and bacterial load of commercial organic egg production farms.

## Figures and Tables

**Table 1:** Results of the Student’s *t*-Test procedure that was carried out for each color background threshold under study. Value of means, standard error (S.E.) and confidence intervals that were obtained are reported.

Color Background Thresholds (∆t)	Mean Values of Colored Pixels (µ*_O.H._* Pixels ± S.E.)	Confidence Intervals of Colored Pixels at 95% (Pixels)	Significance
1	4879 ± 203	4480–5277	*p* < 0.01
3	513 ± 20	473–552	*p* < 0.01
5	132 ± 3	126–138	*p* < 0.01

**Table 2:** Values of sensitivity and specificity that the developed detection sensor showed at the thresholds of color background (CB) evaluated.

Color Background Thresholds (∆t)	Sensitivity (%)	Specificity (%)	Corresponding Cut-off Level (Pixels)
1	80.04	63.55	1763
3	80.22	94.75	196
5	80.14	69.46	74

**Table 3:** Monitoring system accuracy showed in the hens’ detection.

	Positive	Negative	Total
**True**	864	393	1257
**False**	21	18	39
**Total**	885	411	1296
